# Epigenetic regulation in colorectal cancer: The susceptibility of microRNAs 145, 143 and 133b to DNA demethylation and histone deacetylase inhibitors

**DOI:** 10.1371/journal.pone.0289800

**Published:** 2023-08-10

**Authors:** Aadilah Omar, Drishna Govan, Clement Penny

**Affiliations:** Department of Internal Medicine, Oncology Division, Faculty of Health Sciences, University of the Witwatersrand, Parktown, Johannesburg, Republic of South Africa (RSA); University of East Anglia, UNITED KINGDOM

## Abstract

Globally, colorectal cancer (CRC) is a major health concern. Despite improvements in CRC treatment, mortality rates remain high. Genetic instability and epigenetic dysregulation of gene expression are instigators of CRC development that result in genotypic differences, leading to often variable and unpredictable treatment responses. Three miRNAs, miR-143, -145 and -133b, are most commonly downregulated in CRC and are proposed here as potential tumour suppressors. Although the downregulation of these miRNAs in CRC is largely unexplained, epigenetic silencing has been postulated to be a causative regulatory mechanism. Potential epigenetic modulation of miRNA expression, by means of histone acetylation and DNA methylation, was assessed in this study by treating early (SW1116) and late stage (DLD1) CRC cells with the DNA demethylating agent 5-aza-2’-deoxycytidine (5-Aza-2’C) and the histone deacetylase (HDAC) inhibitor Trichostatin A (TSA), respectively. Subsequent quantification of miRNA expression revealed that while all the selected miRNAs were susceptible to DNA demethylation in early- and late-stage CRC cells, susceptibility to DNA demethylation was significantly pronounced in late-stage DLD1 cells. Conversely, although histone acetylation moderately affected miRNA expression in early-stage CRC, it had a marginal effect on the expression of miRNAs in late-stage CRC cells. Overall, this study provides further understanding of the contribution of epigenetics to the regulation of putative tumour suppressor miRNAs in CRC.

## Introduction

As colorectal cancer is among the top three cancers in South African men and women, with a high mortality rate, it poses a significant health concern [1, 2]. CRC is known to develop in an age-dependent, multistep progressive manner, which is characterized by the accumulation of genetic mutations and epigenetic aberrations in response to environmental and other external factors. This process, which involves the transformation of normal colorectal epithelium through progressive steps to form invasive and metastatic cancer, is termed the ‘Adenoma to Carcinoma Sequence’ [3]. This transformation process advances through one of three classical pathways of genomic instability: MSI, chromosomal instability (CIN), and CpG island methylator phenotype (CIMP) [4]. The ‘adenoma-to-carcinoma’ sequence is influenced by epigenetic alterations. Epigenetics provides an additional dimension of gene regulation to the standard gene regulatory networks in a cell, demonstrating the effects of chromatin structure and the external environment on gene expression. Epigenetic mechanisms are involved in the fine-tuning of cellular processes that are critical for proper cell function. Aberrations in these mechanisms ultimately lead to cell dysfunction and are associated with various disease states, including malignant transformation [5].

It should be emphasized that the process of tumorigenesis is a result of the combined influence of multiple epigenetic events. One notable example is the repression of tumour suppressor genes, which often occurs due to the methylation of DNA CpG islands in conjunction with hypoacetylated and hypermethylated histones [6]. The advancement of various drugs targeting epigenetic regulators has facilitated the application of epigenetic-targeted therapy in the treatment of haematological malignancies, demonstrating promising therapeutic potential for solid tumours in both preclinical and clinical trials [7]. While further research is required to elucidate more specific mechanisms, it is widely acknowledged that epigenetic events play a significant role not only in normal biological processes but also in the development of tumours, where alterations in the epigenetic status are frequently observed during cancer initiation. Epigenome-targeted therapy emerges as a highly promising approach for combatting cancer. Given the intricate nature of this disease, epigenetic alterations exert profound effects on various aspects of cancer, including the expression of oncogenes and tumour suppressor genes, as well as signal transduction pathways, ultimately leading to heightened cancer growth, invasion, and metastasis. Although remarkable outcomes have been achieved with epigenetic therapy in haematological malignancies, its effectiveness in solid tumours has been limited, likely due to the distinct characteristics of haematological malignant cells and solid tumour cells, respectively. Nevertheless, researchers continue to explore suitable strategies for solid tumours. Since epigenetic alterations influence the sensitivity of small molecule targeted therapy, chemotherapy, and radiotherapy, epigenetic-targeted therapy holds potential as a valuable adjunctive treatment modality. Furthermore, investigations into the combination of epigenetic therapy with immunotherapy have been conducted in preclinical and clinical trials, demonstrating great promise as a strategy for the treatment of cancer.

DNA methylation is a widely studied epigenetic mechanism that inhibits gene transcription. A general decrease in the load of 5-methylcytosine or hypomethylation is frequently observed in cancer [8]. DNA methylation is concentrated on large stretches of DNA in cancer cells [9]. Thus, it has been determined that these abnormal concentrations of methylated DNA are responsible for silencing classical tumour suppressor genes by aberrant CpG-island-promoter hypermethylation. This phenomenon frequently occurs in various cancer types including CRC [10]; and is also associated with the CIMP pathway [11].

Histone modification is another important epigenetic mechanism that is subject to deregulation in cancer [12]. HDAC 1, 2, 3, and 8 are overexpressed in CRC compared with normal colon epithelium [13]. Consequently, treatment of CRC cells with a histone deacetylase inhibitor (HDACi) induces anti-tumour effects by reducing proliferation signals and inducing apoptosis [14].

In addition to epigenetic alterations, microRNAs (miRNAs), which are non-coding RNAs with epigenetic functions, are important pathways with deregulated patterns in several cancers [15]. miRNAs regulate crucial physiological processes such as development and cellular processes, including cell differentiation, proliferation, and apoptosis [16]. Aberrant expression of these regulators has been associated with carcinogenesis, whereby altered miRNA expression profiles have been identified between normal tissues and derived tumour samples, demonstrating the potential of several miRNAs as putative oncogenes or tumour suppressor genes [17, 18].

Tumour suppressor miRNAs were of particular interest in this study, as their predicted targets would disclose potential oncogenes of clinical significance. miRNA profiling of human CRC revealed several deregulated miRNAs [19]. These studies have identified putative tumour suppressor miRNAs that contribute to colorectal neoplasia [20]. The focus of this study will be on putative tumour suppressor miRNAs -143, -145, and -133b. In this regard, Michael et al. first identified the downregulation of miR-145 and miR-143, in colorectal neoplasia [21]. Subsequently, over fifty CRC-specific differentially expressed miRNAs, that included miR-143 and miR-145, were described [22]. MiR-143 and miR-145 are cluster miRNAs possessing similar gene expression patterns and thus are therefore commonly reported together, especially with regard to tumour suppressor activity in CRC [22–25]. MiR-133b has also been reported to act as a tumour suppressor and exhibits decreased expression in CRC according to several reports [26–29]. As miR-145, miR-143 and miR-133b seem to commonly be downregulated in CRC, the hypothesis was made that ultimately the combination of the three miRNAs would affect pathways related to tumorigenesis and potentially in CRC development. Under this inference the targets identified per miRNA were used to generate a KEGG pathway enrichment of the combined miRNA predicted targets (See S1 File).

Because of the tumour suppressor nature of miR-143, -145, and -133b, coupled with the known fact that tumour suppressors are aberrantly silenced through epigenetic mechanisms in cancer, it is postulated here that these miRNAs are regulated in the same manner. Furthermore, elucidation of miR-143, -145, and -133b gene targets could reveal potential oncogenes of therapeutic relevance. This study aimed to assess the epigenetic alterations by means of DNA methylation and histone acetylation on the expression of three putative tumour suppressor miRNAs in CRC cell lines. In the present study, cell lines from two different progressive stages (SW116 early, DLD1 late) of CRC were utilized to assess the stage-specific alterations in miRNA expression post treatment with the epigenetic agents 5-Aza-2’-C and TSA.

## Materials and methods

### Ethical clearance

An ethics waiver (Ref: W-CJ-090317-4) was granted by the Human Research Ethics Committee (HREC Medical) of the University of Witwatersrand, Johannesburg, stating that the following research does not require clearance.

### Cell culture and conditions

SW1116 (ATCC CCL-233™ Human colorectal adenocarcinoma, stage I, RRID: CVCL_0544) and DLD1 (ATCC CCL-221™ Human colorectal adenocarcinoma, stage III (metastatic), RRID:CVCL_0248) cells were obtained from the ATCC. These cells were routinely cultured in DMEM-F12 (Lonza-Biowhittaker®) supplemented with 10% heat-inactivated foetal bovine serum and 1% penicillin/streptomycin (Lonza BioWhittaker®). Cells were incubated at 37°C in an atmosphere of 5% CO_2_ in air.

### Epigenetic drug treatments and cell viability

SW1116 and DLD1 cells were subjected to epigenetic treatments involving two entities of epigenetic regulation: DNA methylation and histone acetylation. For cell synchronicity, cultures were serum starved overnight and then treated with 5-Aza-2’-C (Sigma Aldrich) at concentrations of 1μM and 3μM (dissolved in 99% acetic acid:PBS), respectively, and incubated for 48 h with a daily replacement of the drug due to the instability of the 5-Aza-2’-C compound. Cells were also treated with TSA (Sigma-Aldrich) (reconstituted in DMSO) to assess the effects of histone acetylation on miRNA expression. After serum-starving overnight, cells were subjected to a 24-hour treatment with 300nM TSA. A DMSO (Sigma-Aldrich) carrier control was included in the treatments to evaluate non-specific effects of this diluent. Post-treatment cells were harvested and re-suspended in 1mL PBS mixed with 1mL sterile filtered Trypan Blue (Bio-Rad) and incubated for 3 min at room temperature. Thereafter, 100μL of the Trypan Blue/cell suspension was placed on a haemocytometer slide, viewed and counted using an inverted bifocal microscope. The cell viability was calculated using the following equation:

$$\%\;viable\;cells=\frac{Total\;number\;of\;viable\;cells\;per\;1\;mL\;of\;aliquot}{Total\;number\;of\;cells\;per\;1mL\;of\;aliquot}\;X\;100$$

### MicroRNA isolation, quantitation, and assessment

Total RNA was purified using the miRvana miRNA isolation kit (Ambion), according to the manufacturer’s guidelines. RNA samples were then stored at -80°C until use. The concentration and purity of the isolated RNA were determined using a Thermo Scientific Nanodrop 1000 Spectrophotometer (RRID:SCR_016517), and the RNA concentrations and A260/A280 and A260/A230 ratios for each sample of the extracted RNA were calculated. Only samples that achieved high levels of purity were used in the subsequent experiments. Samples with an OD A260/A280 ratio of between 1.8 and 2.1 were considered as highly pure RNA.

### miRNA reverse transcription

Conversion of extracted total RNA to cDNA was achieved using the miRNA Reverse Transcription kit (Applied Biosystems), using miRNA-specific stem loop primers for miR-143, -145 and -133b, respectively. RNA concentrations were standardized for all samples before the Reverse Transcription assay. The RT Master mix was centrifuged and separated into 7μL aliquots. Five microliters of total RNA at a standardized concentration was then added, and subsequently, 3μL of the miRNA RT primers were added to each aliquot, adding up to 15μL reaction volumes. Reaction mixtures were kept on ice for 5 min and then transferred to a BIO-RAD MJ Mini™ Personal Thermal Cycler under the following cycling conditions: hold for 30 min at 16°C, hold for 30 min at 42°C, hold for 5 min at 85°C, and hold at 4°C. MiRNA expression was normalised to the housekeeping non-coding RNA 18s rRNA and detected relative to no treatment controls. The RT reaction for the amplification of 18s rRNA did not contain specific reverse transcription primers, and hence was not performed using the miRNA reverse transcription kit. The RT procedure was carried out with a conventional TaqMan® Reverse Transcription kit using random hexamers as primers to convert total RNA to cDNA.

### miRNA PCR amplification

Real-time PCR was performed using the TaqMan® Universal PCR Master Mix and TaqMan® MicroRNA Assays (Applied Biosystems). miRNA expression was normalized to the housekeeping non-coding RNA 18s rRNA and detected relative to no-treatment controls in an Applied Biosystems 7500 Real-Time PCR System (RRID:SCR_018051). Input cDNA concentrations were standardized prior to the experimental runs on each cell line. miRNA specific TaqMan MGB probes and primers were used to detect the expression of the respective miRNAs. Samples were run in triplicate to establish the mean Ct value for the amplification of each sample. The housekeeping non-coding RNA 18SrRNA was amplified using an assay containing specific primers and probes targeting this gene. The parameters programmed into the 7500 Real Time PCR machine for the AmpliTaq Gold Enzyme Activation included a 10-minute hold step at 95°C. PCR ran for 40 cycles with 15s denaturing step at 95°C and 60s annealing/extending step at 60°C.

### Data analysis

The sample reactions were performed in triplicates. The mean Ct values were determined during the experimental runs, and subsequently, the miRNA expression levels from the treated samples were first normalised to 18SrRNA and then calculated relative to no treatment controls, according to the 2^-ΔΔCt^ method described by Livak and Schmittgen in 2001 [30]. To compare the relationship between the sample means before and after epigenetic treatments, a paired two-tailed Student’s t-test was performed with the confidence interval set at 95%.

## Results

### The effect of DNA demethylation on cell viability in SW1116 and DLD1 cells

To evaluate the susceptibility of SW1116 and DLD1 cell lines to 5-Aza-2’-C treatment, the cell lines were subjected to cell viability assays post-treatment with the epigenetic drug and compared to the untreated controls. Visual changes observed in SW1116 cells after epigenetic treatment with 5-Aza-2’-C resulted in a marginal increase in cell growth rate (results not shown). This effect was observed as the cell cultures reached confluence at a slightly faster rate. After treatment with 5-Aza-2’-C, passage cycles were shorter than those of untreated SW1116 cell cultures. In contrast, following treatment with 5-Aza-2’-C, the altered growth characteristic of DLD1 cells was demonstrated by a slight decrease in adherent cell growth. This was confirmed by the detachment of several cells from the cell culture flask surfaces. The detached cells lost their epithelial morphology and appeared more granular.

**Fig 1:**
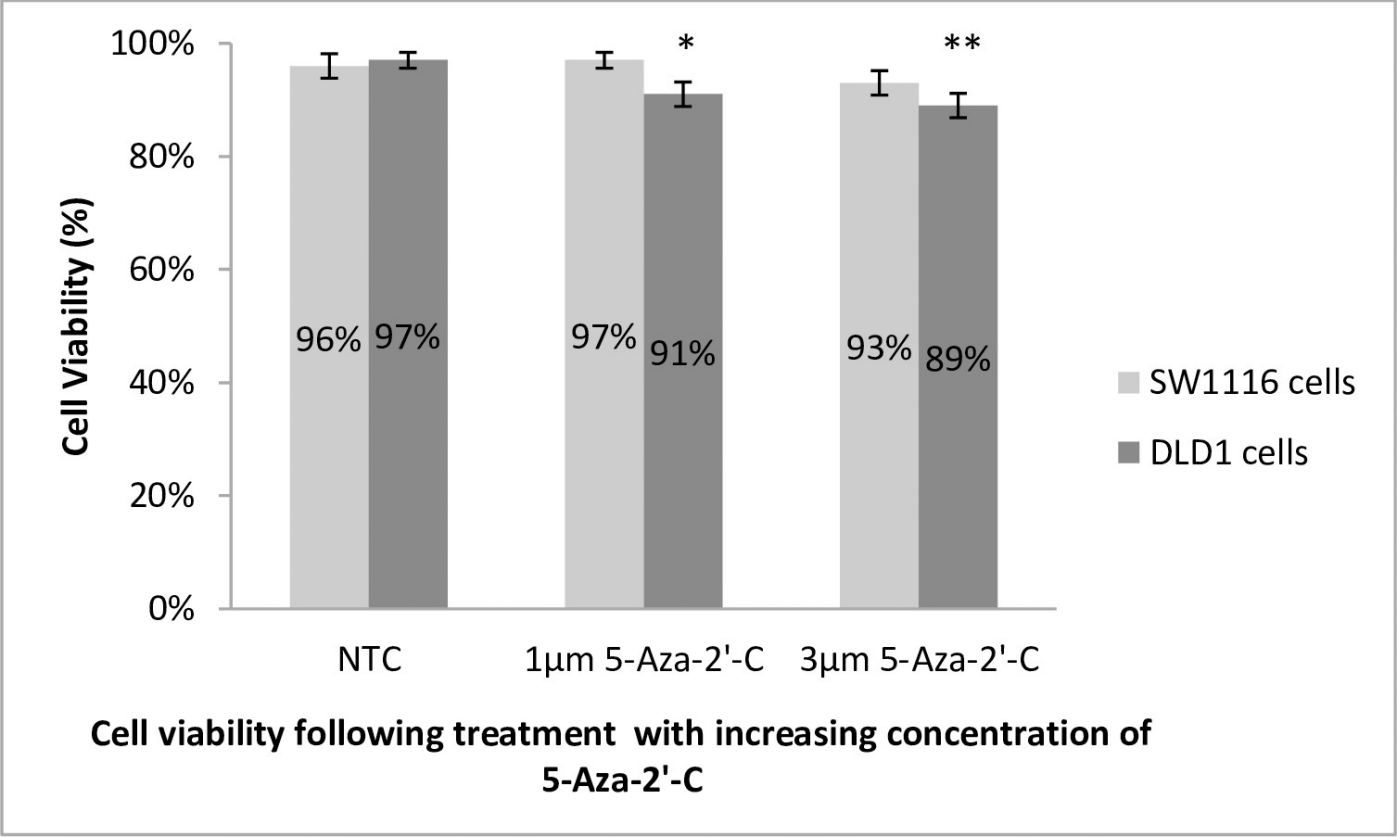
SW1116 and DLD1 cell viability following 5-Aza-2’-C treatment. Cell viability assays conducted on SW1116 cells following treatment with 5-Aza-2’-C correlated with the visual changes noted with the number of live cells increasing by 1% to a level of approximately 97% viability when treated with 1μm 5-Aza-2’-C (p = 0.5387), compared to 96% viability for the untreated equivalent of the cells. However, with an increased concentration (3μm) of the DNA demethylating agent, cell viability decreased with a 3% drop in viability to 93%, compared to the untreated equivalent (p = 0.1642). The “no treatment control” (NTC) samples for the DLD1 cell line yielded a 97% cell viability while treatment with 1μM 5-Aza-2’-C decreased cell viability by 6% yielding a value of approximately 91% live cells (p = 0.0157). The number of live cells were further decreased to 89% at the increased dose of 3μM 5-Aza-2’-C (p = 0.0058). Overall, the effect of 5-Aza-2’-C was most notable in the late-stage cancer cell line DLD1. * Significant (p<0.05); ** Very significant (p<0.01).

Cell viability assays conducted on SW1116 cells pre- and post- treatment with 5-Aza-2’-C correlated with the visual changes observed. A negligible increase in viability was observed with treatment with 1μm 5-Aza-2’-C (p = 0.5387); however, with an increased concentration (3μm) of the DNA demethylating agent, cell viability decreased to 93% (p = 0.1642), compared to the untreated equivalent. Treatment with 1μM 5-Aza-2’-C decreased cell viability to 91% (*p = 0.0157) in DLD1 cells, with a further significant decrease to 89% (**p = 0.0058) at the higher dose of 3μM 5-Aza-2’-C (Fig 1).

### The effect of histone acetylation on cell viability in SW1116 and DLD1 cells

Microscopic assessment of the effect of TSA on SW1116 cells revealed an increase in non-adherent cells and altered cell viability. More specifically, the cells in the suspension were granular in appearance. Similarly, DLD1 cells after treatment with 300nM TSA showed a noticeable increase in non-adherent cells (results not shown).

**Fig 2:**
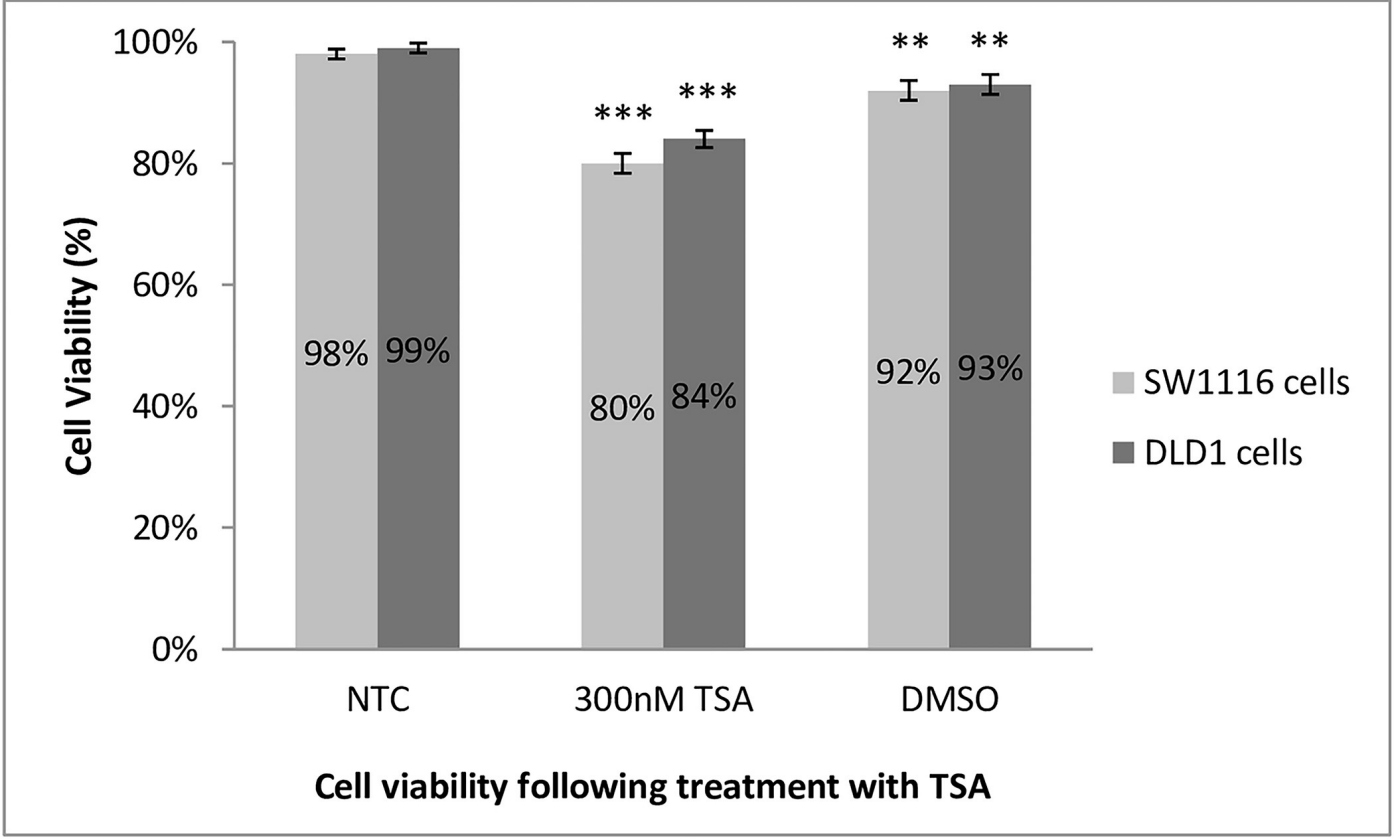
SW1116 and DLD1 cell viability following TSA treatment. Consistent decreased viability was demonstrated in SW1116 cell cultures after treatment with 300nM TSA. The dramatic percentage decrease in viable cells compared to the untreated control cells was 18% (p = 0.0001). This drop was independent of DMSO as the DMSO treated control cells had diminished the percentage of live cells by 6% (p = 0.0047). Similarly, the cell viability assay of DLD1 cells showed untreated cells to be 99% viable, whilst DMSO carrier control cells had a minimally decreased viability of some 6% (p = 0.0047). TSA treatment in comparison, resulted in a 15% decrease in DLD1 cell viability (p = 0.0001). ** Very significant (p<0.01), *** Extremely significant (p<0.001).

DMSO carrier controls were included to ascertain the possible effect on the viability of cells and to differentiate them from the effects of TSA. The DMSO control influenced the viability of both SW1116 (**p = 0.0047) and DLD1 cells (**p = 0.0001). A uniform pattern of decreased viability was demonstrated in SW1116 cell cultures after treatment with 300nM TSA with an extremely significant decrease in viable cells to 80% compared to untreated control cells (***p = 0.0001). TSA treatment also resulted in a significant decrease in cell viability of 84% in the DLD1 cell line (***p = 0.0001) (Fig 2). In both instances, the effect of DMSO was noted; however, TSA reduced cell viability to a greater extent.

**Fig 3:**
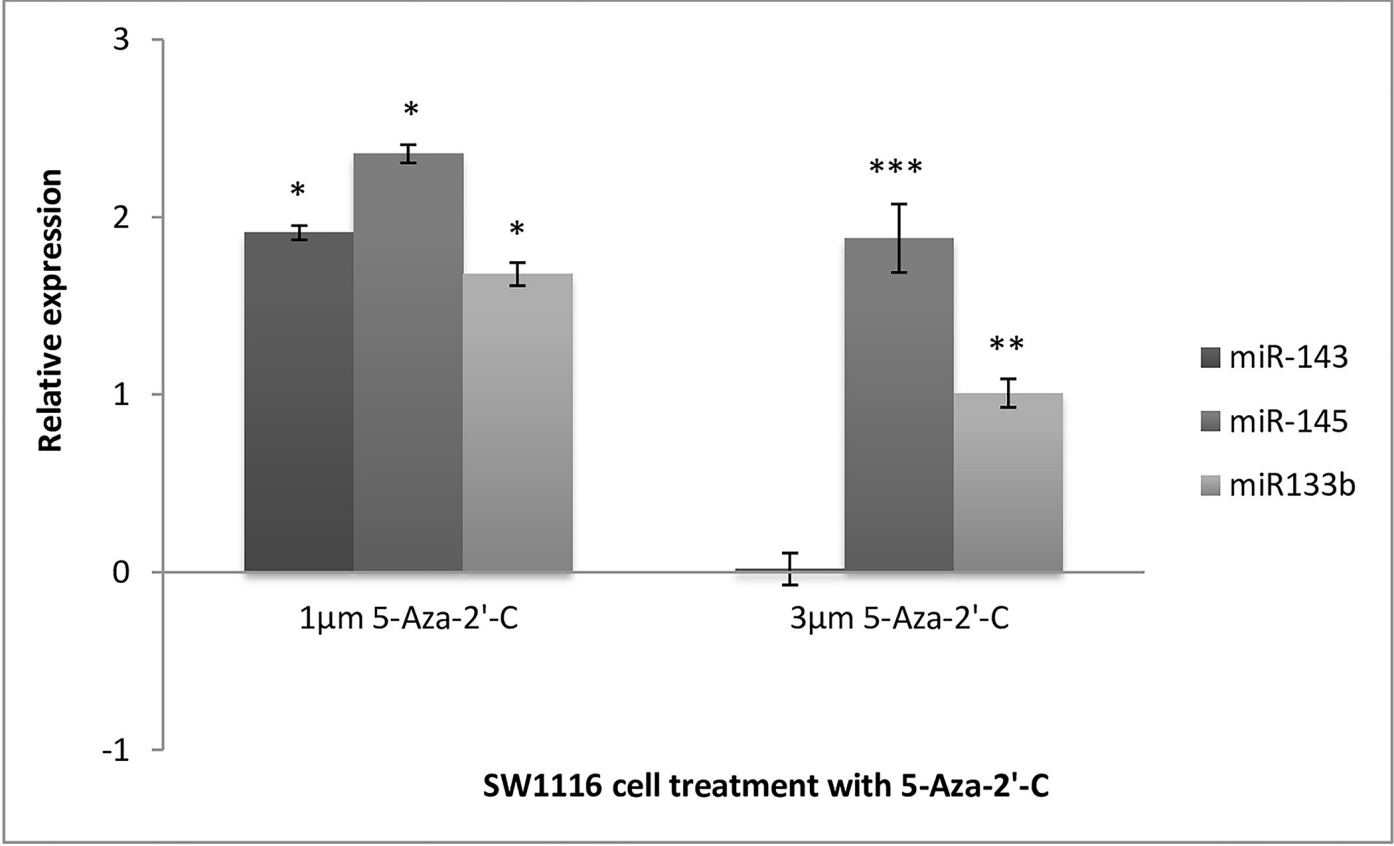
Relative expression of miR-143, miR-145 and miR-133b in SW1116 cells after treatment with 5-Aza-2’-C. Low dose (1μm) 5-Aza-2’-C yielded increased miR-143 expression by almost 2-fold (p = 0.0157), while there was a marginal increase of 0.018-fold (p = 0.503) after high dose (3μm) 5-Aza-2’-C treatment. Relative expression of miR-145 in SW1116 cells after treatment with 5-Aza-2’-C. Low dose (1μm) and high dose (3μm) 5-Aza-2’-C yield increased miR-145 expression by 2.4-fold (p = 0.0177) and 1.8-fold (p = <0.00001) respectively. Relative expression of miR-133b in SW1116 cells after treatment with 5-Aza-2’-C. Low and high dose 5-Aza-2’-C treatment yield increased miR-133b expression by 1.6 (p = 0.0282) and 1-fold (p = 0.0047) respectively. *Significant (p<0.05), ** Very Significant (p<0.01), *** Extremely significant (p<0.001).

**The effect of DNA demethylation on miRNA expression in SW1116 and DLD1cell lines.** To evaluate the effect of DNA de-methylation on miR-143 expression in early-stage CRC cells, the expression levels of miR-143 relative to the untreated controls were compared after treating SW1116 cells with low and high doses of 5-Aza-2’-C for 48-hours (Fig 3). The Ct values were first normalized to the endogenous control, 18s rRNA, after which fold changes in expression were calculated according to the 2^-ΔΔCt^ method. The expression of miR-143 was effectively induced by treatment with 1μm 5-Aza-2’-C by almost 2-fold (*p = 0.0157). However, at a higher concentration (3μm) this induced expression was lost, demonstrating a marginal but insignificant upregulation of 0.018-fold (p = 0.503), compared to the untreated control. At the lower dose of 1μm 5-Aza-2’-C, miR-145 was significantly up-regulated by 2.4-fold (*p = 0.0177), relative to the control cells; the mean decrease in the Ct value was significant. Similarly, at a dose of 3μm 5-Aza-2’-C, miR-145 was effectively upregulated by 1.8-fold (***p<0.00001) compared to the untreated control samples. MiR-133b expression increased by more than 1.6-fold (*p = 0.0282) after treatment with 1μM 5-Aza-2’-C. A similar trend was observed for the treatment of SW1116 cells with the higher dose of 3μm 5-Aza-2’-C; miR-133b was also significantly upregulated (**p = 0.0047). From this, a possible dose-dependent relationship between 5-Aza-2’-C treatment and miRNA expression was deduced.

**Fig 4:**
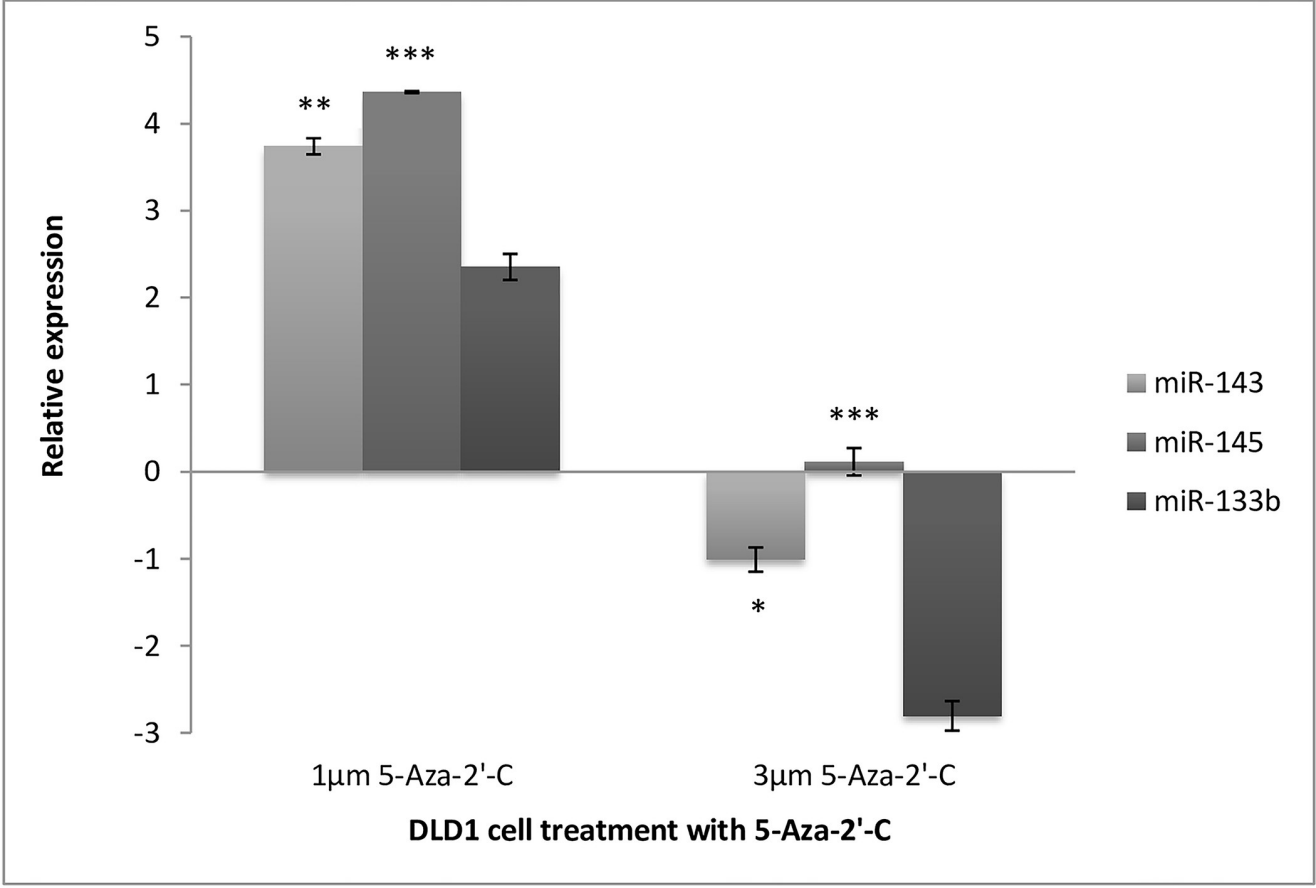
Relative expression of miR-143, miR-145 and miR-133b in DLD1 cells after treatment with 5-Aza-2’-C. Low dose (1μm) yielding an increased expression of miR-143 by 3.7-fold (p = 0.005), while high dose (3μm) 5-Aza-2’-C exhibits down-regulation of miR-143 by 1-fold (p = 0.0238). Relative expression is shown as log (2^-ΔΔCt,^ base 2). A Low dose of 5-Aza-2’-C (1μm) yielded an increased expression of miR-145 by 4.3-fold (p = 0.001) while high dose (3μm) 5-Aza-2’-C exhibited marginal increase of miR-145 by 0.1-fold (p = 0.00001). Relative expression is shown as log (2^-ΔΔCt^, base 2). Treatment with 5-Aza-2’-C (1μm) yielded an increased expression of miR-133b by 2.3-fold (p = 0.1179, not significant) while a higher dose (3μm) of 5-Aza-2’-C exhibits down-regulation of miR-133b by 2,8-fold (p = 0.0870, not significant). Relative expression is shown as log (2^-ΔΔCt, base^ 2). *Significant, (p<0.05), ** Very Significant (p<0.01), *** Extremely significant (p<0.001).

MiR-143 expression was assessed in DLD1 cells after treatment with 1μm and 3μm doses of 5-Aza-2’-C (Fig 4). Quantification of miR-143 revealed that when treated with the lower dosage, miR-143 was significantly upregulated by 3.7-fold (**p = 0.005). In contrast, treatment with a higher dose of 3μm resulted in the downregulation of miR-143 (*p = 0.0238). When comparing these results with the quantitative results of the effect of 5-Aza-2’-C on miR-143 in the early-stage CRC cell line SW1116 (Fig 3), it was immediately recognized that in both cell lines, miR-143 is more susceptible to 1μm 5-Aza-2’-C than the higher dose of 3μm. Assessment of the relative expression of miR-145 to the untreated controls in DLD1 cells after treatment with 1μm 5-Aza-2’-C revealed that it was effectively upregulated by 4.3-fold relative to the untreated controls (***p = 0.001). In contrast, when DLD1 cells were treated with 3μm 5-Aza-2’-C, a slight increase in the expression of miRNA was apparent, which was found to be significant (***p = 0.00001). As shown in Fig 4, relative expression of miR-133b compared to untreated controls showed a trend of upregulation of 2.3-fold when treated with 1μm 5-Aza-2’-C (p = 0.1179). Alternatively, a trend of down-regulation of miR-133b by 2.8-fold is evident when DLD1 cells were treated with a higher dose of 5 Aza-2’-C (p = 0.0870). This could possibly imply a dose-sensitive response of miR-133b to the DNA demethylating agent. In comparison to Fig 3, early-stage colorectal carcinoma cells (SW1116) were treated with 5-Aza-2’-C and the expression of miR-133b was evaluated. miR-133b expression in SW1116 cells was more susceptible to DNA 5-Aza-2’-C at a higher dose of 3μm than in DLD1 cells. In contrast, susceptibility to the 1μm dose was retained in both cell lines, with the highest susceptibility observed in the DLD1 cell line.

### The effect of histone acetylation on miRNA expression in SW1116 and DLD1 cell lines

**Fig 5:**
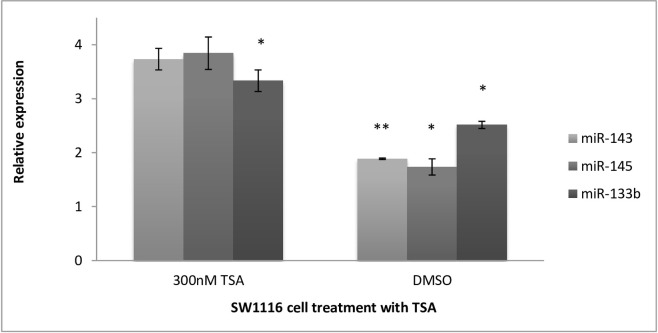
Relative expression of miR-143, miR-145 and miR-133b in SW1116 cells after treatment with TSA. It was determined that relative to the no treatment controls, when the cells were treated with 300nM TSA, miR-143 had increased in expression by 3,7-fold (p = 0.0759). DMSO alone only increased the expression of miR-143 by 1.8-fold, almost 1.9-fold less than that of 300nM TSA. The quantification of miR-143 post treatment with DMSO was found to be significant (p = 0.0028). The effect on the expression of miR-145 was much like that of miR-143. Treatment with 300nM TSA resulted in a mean decrease in Ct (p = 0.0508), providing evidence that treatment with TSA demonstrates a trend of up-regulation of miR-145 by 3.8-fold, relative to the no treatment control. DMSO treatment had induced the expression of miR-145 by 1.7-fold, (p = 0.0332). TSA significantly increased the expression of miR-133b by 3.3-fold, (p = 0.0353). DMSO had induced a significant increase in miR-133b expression by 2.5-fold (p = 0.0101). Although DMSO had also induced expression of the miR-133b, this was 0.8-fold less than the expression for miR-133b after treatment with 300nM TSA. Relative expression is shown as log (2^-ΔΔCt^, base 2), where p<0.05 is significant.

The effect of histone acetylation on the expression dynamics of miR-133b in early-stage colorectal adenocarcinoma was assessed by treating SW1116 cells with 300nM TSA and quantitatively determining the expression by qRT-PCR (Fig 5). miR-143 expression increased by 3,7-fold (p = 0.0759) when treated with TSA. Interestingly, DMSO significantly increased the expression of miR-143 by 1.8-fold (p = 0.0028). A similar expression pattern was observed with miR-145, where treatment with 300nM TSA resulted in a mean decrease in Ct. This provides evidence that treatment with TSA demonstrates a trend of upregulation of miR-145 by 3.8-fold (*p = 0.0508) relative to the no-treatment control. DMSO treatment alone induced the expression of miR-145 by 1.7-fold (p = 0.0332). This result indicates a net upregulation of miR-145 after treatment with 300nM TSA by approximately 2-fold compared to the untreated controls. The histone deacetylase inhibitor significantly increased the expression of miR-133b by 3.3-fold (*p = 0.0353). As shown in Fig 5, DMSO had induced a significantly increased miR-133b expression by 2.5-fold (*p = 0.0101). Although DMSO also induced the expression of miR-133b, it was 0.8-fold lower than the expression of miR-133b after treatment with 300nM TSA.

**Fig 6:**
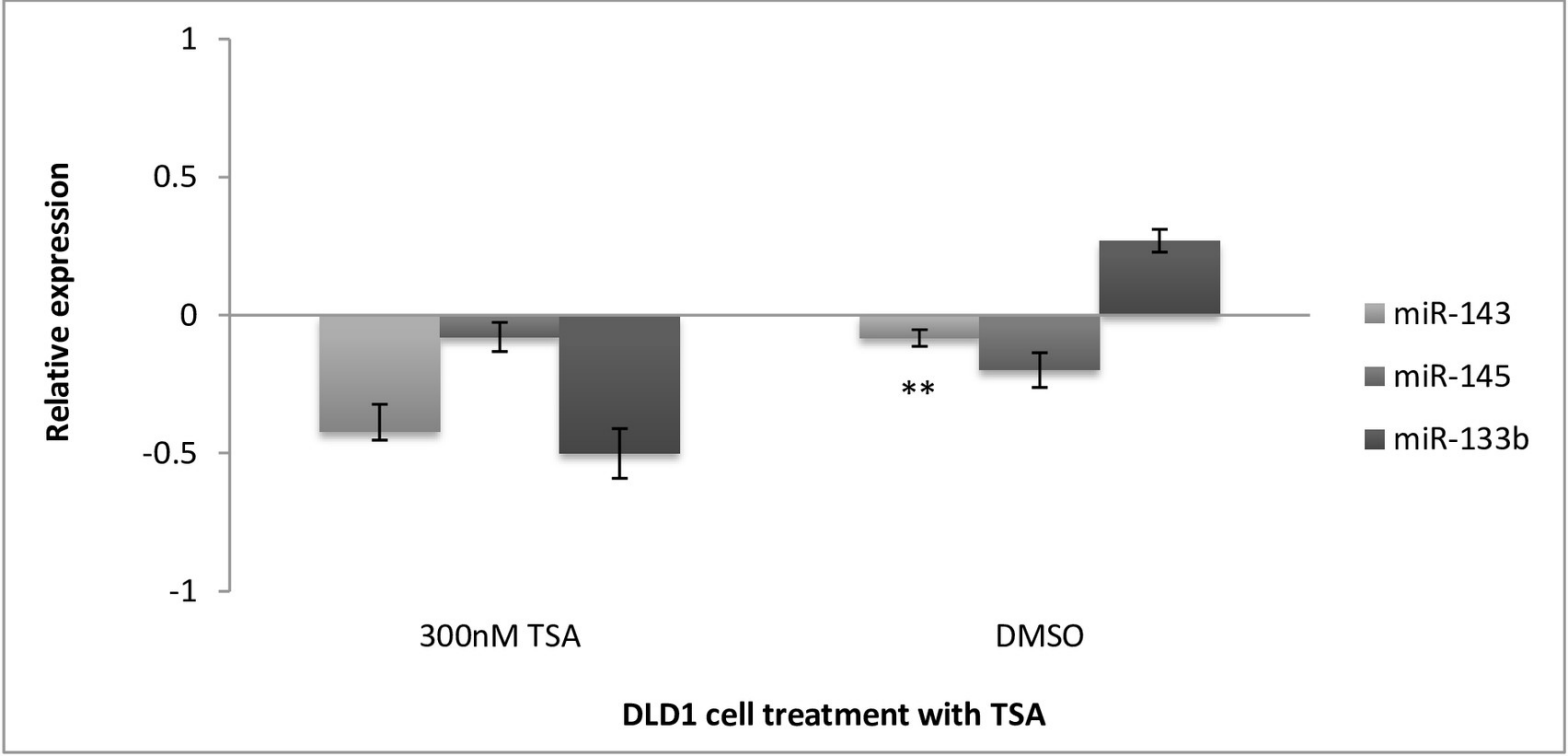
Relative expression of miR-143, miR-145 and miR-133b in DLD1 cells after treatment with TSA. miR-143 had decreased by 0.4-fold relative to the no treatment. This mean increase in Ct was found to be non-significant (p = 0.3277). Treatment with DMSO alone had significantly decreased miR-143 expression by 0.08-fold, (p = 0.007515). MiR-145 expression in late-stage colorectal adenocarcinoma was found to be mildly affected by 300nM TSA. The mean Ct was slightly increased compared to the NTC (p = 0.6591). Therefore miR-145 expression decreased slightly by 0.07-fold, relative to the NTC. The DMSO control exhibited a mean increase in Ct (p = 0.3383) with an almost 0.2-fold decrease in expression was noted when treated with DMSO. A decrease in miR-133b expression by 0.5-fold from the NTC was seen (p = 0.32). However, the DMSO alone had demonstrated a mean decrease in Ct, (p = 0.1171), showing increased expression of miR-133b by 0.26-fold. Relative expression is shown as log (2-^ΔΔCt,^ base 2). ** Very Significant (p<0.01).

The effect of histone acetylation in late-stage CRC was assessed by treating DLD1 cells with 300nM TSA and the DMSO carrier control, after which the three miRNAs in question, miR-143, 145 and 133b, were quantified by miRNA qPCR relative to the untreated controls (Fig 6). MiR-143 expression in late-stage CRC after treatment with 300nM TSA revealed that the epigenetic drug decreased the expression of miR-143 by 0.4-fold relative to the no-treatment control (p = 0.3277). Treatment with DMSO alone significantly decreased miR-143 expression by 0.08-fold compared with the untreated control (**p = 0.007515). It can be deduced that TSA had a greater effect on the expression of miR-143 than DMSO. MiR-145 expression in late-stage colorectal adenocarcinoma was marginally affected by treatment with 300nM TSA. Therefore, miR-145 expression decreased by 0.07-fold (p = 0.6591). Surprisingly, the DMSO carrier control exhibited almost a 0.2-fold (p = 0.3383) decrease in expression. An antagonistic relationship between treatment with TSA and miR-133b was demonstrated, whereby treatment with TSA resulted in a decrease in miR-133b expression by 0.5-fold (p = 0.32). This effect seemed to be independent of the DMSO vehicle, as DMSO had demonstrated an increased in the expression of miR-133b, by 0.26-fold (p = 0.1171).

## Discussion

Colon cancer remains the leading cause of cancer-related deaths worldwide. The consistent pattern of downregulation of miRNAs -143, -145 and -133b in CRC suggests that these miRNAs are potential targets of epigenetic regulation.

### Cell viability

As a first approach, the viability of each cell line was assessed following treatment with 5-Aza-2’-C, since it has previously been shown to suppress growth of tumour cell lines [31, 32]. This indicates the role of DNA demethylation on influencing the *in vitro* growth of early- and late-stage CRC cells. DLD1 cells displayed decreased cell viability in a dose-dependent manner upon treatment with 5 aza-2’-C. However, in SW1116 cells, although there was a marginal increase in the percentage cell viability at the lower dose of 1μm 5-Aza-2’-C, there was nevertheless a slight decrease in cell viability at the higher dose (3μm) of 5-Aza-2’-C.

The lack of susceptibility of SW1116 cells to 5-Aza-2’-C is inconsistent with published literature reporting the antitumour effects of 5-Aza-2’-C on other early-stage CRC cell lines [33]. However, the mechanisms by which 5-Aza-2’-C accomplishes its anti-tumour effects have not been fully elucidated. The first postulated mechanism of cytotoxicity is related to the reactivation of aberrantly silenced genes that ultimately control and regulate cell proliferation and apoptosis. The second idea relates to treatment with 5-Aza-2’-C, which is recognized as DNA damage due to the covalent DNMT-DNA adducts that form, following which the DNA damage response pathways are responsible for the cytotoxic effects [34].

A key difference between the two cell lines is that DLD1 cells are microsatellite unstable (MSI), whereas SW1116 cells are microsatellite stable (MSS) [35]. SW1116 cells are MSS, indicating the presence of unstable chromosomal translocations with gains or losses of whole chromosomes [36, 37]. Generally, this transient instability of the chromosomes occurs early in the development of cancer, which is likely the case as SW1116 represents Dukes’ stage A (early) or Stage I of CRC.

TSA treatment at a concentration of 300nM for 24 h potently decreased the viability of both early (SW1116) and late (DLD1) stage CRC cell lines. These results were not unexpected, as HDACi have long been known to induce cell cycle arrest and cell death in cancer cells [38–40].

In the present study, when comparing the effect of DNA demethylation *versus* that of histone deacetylase inhibition on cell viability in both cell lines, it is evident that TSA decreases cell viability in both cell types more than DNA demethylation, possibly because various cell death pathways are initiated.

### Stage specific differences in miRNA expression changes upon DNA methylation

The results demonstrated a dose-dependent response to the DNA demethylating agent 5-Aza-2’-C. MiR-145 and miR-143 are polycistronic miRNAs, indicating that they are transcribed from the same promoter [41]. In the present study, although both miRNAs were induced by 1μM 5-Aza-2’-C, they were at varying levels. These differential levels of induction of both miRNAs by 5-Aza-2’-C could be explained by the post-transcriptional regulation of the miRNA maturation process. MiR-133b expression, also induced by 5-Aza-2’-C in this study in both cell lines at the lower dose and only in SW1116 cells at the higher dose, correlates with a recent finding, whereby miR-133b promoter hypermethylation was reported to be causal for its low levels in CRC [41].

As described above, the adenoma-to-carcinoma sequence alludes to the accumulation of genetic and epigenetic alterations in a stepwise manner, for the transformation from normal colon epithelium to adenocarcinoma [2]. More specifically, the accumulation of DNA methylation during the transformation process was highlighted in this model. Thus, it follows that DLD1 late-stage CRC cells have a higher degree of DNA methylation than SW1116 cells. This could explain the tendency of miRNAs to be more highly induced in DLD1 cells than in SW1116 cells, when treated with the DNA demethylation agent.

Furthermore, all miRNAs were more responsive to the lower dose of 5-Aza-2’-C in DLD1 cells than in SW1116 cells. For miR-145 this could be explained by the discrepancy in promoter hypermethylation at different stages, as indicated by the lack of promoter hypermethylation in SW1116 cells and the presence of miR-145 methylation in several Dukes stage C/p53 mutant CRC cell lines [42]. Another potential reason is that 5-Aza-2’-C is an S-phase acting drug, which means that it induces its effect in actively replicating cells. SW1116 is a slow-growing cell line with a doubling time higher than that of DLD1 cells. Thus, it may be assumed that SW1116 cells have a proportionately lower ratio of cells in the S phase than DLD1 cells at a given time point. This would need to be confirmed by measuring the methylation levels of each cell line before and post- treatment with 5-Aza-2’-C.

When comparing the responsiveness of each miRNA to the DNA demethylating agent, it is evident that miR-145 is most susceptible to DNA demethylation, followed by miR-143, and then miR-133b. This pattern is consistent regardless of the stage of the cancer cell line; therefore, it may be assumed that miRNA regulation occurs in the same manner in both early and late-stage CRC cells.

### Stage specific differences in miRNA expression changes upon histone deacetylation

In early-stage CRC cells, all three miRNAs were induced following TSA treatment. In comparison, TSA did not seem to induce miRNA expression in the late-stage CRC cell line, showing a slight downregulation. These results suggest that the susceptibility of these miRNAs to HDACi is dependent on the representative tumour stage of the cell line.

In studies where tumours were treated with HDACi’s, researchers became aware of a phenomenon in which cells would acquire resistance to HDACi [43]. Notably, resistance to TSA in colon cancer cells was caused specifically by a deficiency in the MLH1 gene, as acquired resistance was only present in MSI cells [44]. This could potentially explain why TSA had no effect on miRNA expression in the present study.

### Comparison of DNA demethylation versus histone acetylation on expression of miRNAs

TSA showed comparable induction of miRNAs to 5-Aza-2’-C-treated cells in the early-stage CRC cell line SW1116. However, when evaluating the effect of DNA demethylation compared to histone acetylation in late-stage CRC, there was a marked difference in the transcriptional induction of all three miRNAs. DNA methylation appears to play an influential role in the regulation of miRNAs in the later stages of CRC. In contrast, histone acetylation plays a moderate role in early CRC, but does not seem to have a significant effect on late-stage CRC.

## Conclusion

Deregulation of miR-145, -143, and -133b in CRC indicates that these small regulatory RNAs are key drivers of tumorigenesis. Analysis of their regulation and biological targets will provide valuable information for CRC therapeutics. The present study showed that although all the chosen miRNAs could undergo DNA demethylation in both early and late-stage CRC cells, the susceptibility to DNA demethylation was significantly more pronounced in late-stage 3 DLD1 cells. On the other hand, histone acetylation had a moderate influence on miRNA expression in early-stage 1 CRC, but its effect on miRNA expression in late-stage CRC cells was minimal. These connections have been suggested to be linked to genetic variations between the MSS SW1116 cell line and the MSI of DLD1 cells. This insight could be useful in tailoring treatment plans and influence the use of these agents in combination with other therapeutics.

These miRNAs also have potential as predictive markers and therapeutic targets [44]. While DNA demethylating agents have shown potential for treating CRC, the overall response has however been inadequate [45]. Furthermore, their use is tainted by the DNA damage-related side effects. Interestingly, a recent combination with paracetamol resulted in enhanced anti-tumour activity through the induction of oxidative stress [46]. Despite HDACi’s showing promising anti-cancer activity, monotherapy in solid tumours was found to be mostly ineffective. This has resulted in the redirection of the focus towards combined inhibition strategies [47]. This synergistic enhancement has been proven to be successful in conjunction with alkylating agents, proteasome inhibitors, radiotherapy, immunotherapy, and tyrosine kinase pathway inhibitors.

## Supporting information

S1 File (DOCX)

## Abbreviations

18s rRNA: 18s ribosomal ribonucleic acid
5-Aza-2’-C: 5-aza-2’-deoxycytidine
cDNA: Complementary DNA
CIMP: CpG island Methylator Phenotype
CIN: Chromosome Instability
CRC: Colorectal cancer
DMEM-F12: Dulbecco’s Modified Eagle Medium:F-12 Nutrient mixture
DMSO: Dimethyl sulfoxide
DNA: Deoxyribonucleic acid
DNMT: DNA methyltransferase
FBS: Foetal bovine serum
HDAC: Histone deacetylase
HDACi: Histone deacetylase inhibitor
miRNA: microRNA
mRNA: messenger ribonucleic acid
MSI: Microsatellite Instable
MSS: Microsatellite Stable
PBS: Phosphate Buffered Saline
PCR: Polymerase Chain Reaction
qPCR: quantitative polymerase chain reaction
qRT-PCR: quantitative real-time polymerase chain reaction
RNA: Ribonucleic acid
TSA: Trichostatin A
