# Supplementary material for: Epigenetic regulation in colorectal cancer: The susceptibility of microRNAs 145, 143 and 133b to DNA demethylation and histone deacetylase inhibitors
Source: PLoS One. 2023 Aug 10;18(8):e0289800. doi: 10.1371/journal.pone.0289800 (PMC10414600; doi:10.1371/journal.pone.0289800)
Supplement: S1 File — (DOCX) [file pone.0289800.s001.docx]

# Bioinformatics analysis of putative targets

## Computational tool selection

In this study three tools were selected to predict targets for the miRNAs of interest. The tools were selected to encompass the various principles of miRNA-target binding. Also, a key consideration is the sensitivity and specificity of each program which justifies the reliability of the results retrieved through the program. The motivation behind using three different target prediction programs lies upon the hypothesis that if all three programs predict the same target by using different algorithms, then it is more likely to be a true target. Only targets that were predicted by all chosen programs were considered for further filtering. The features of each program are described below:

### Program 1: TargetScan v5.1 (<http://targetscan.org>)

TargetScan utilises a rule-based algorithm that first searches for full seed region complementarity and thereafter extends the search to regions outside the seed region (approximately 21-23 nucleotide fragments) until a mismatch is detected. Classifications are then made on the length of perfect complementarity and an adenine at position 1. Imperfect seed matches with 3’ compensatory pairing are also considered in this algorithm to accommodate G:U wobbles. The complementary regions are then analysed by the RNAfold program to determine the minimum free energy secondary structure. Conservation is considered in this algorithm by aligning orthologous 3’UTRs from up to 5 different species and then determining if the seed region is located in an island of conservation. The resulting score is calculated per 3’UTR. The scoring relies on several parameters; the type of seed matching, any pairing that occurs outside of the seed region, AU content upstream and downstream of the seed region and the overall distance of the site to the nearest untranslated region [1].

### Program 2: PicTar – Probabilistic Identification of Combination of Target sites (<http://pictar.bio.nyu.edu>)

Unlike TargetScan, PicTar is a data-driven algorithm, rather than a rule-based one. This essentially means that prediction of miRNA targets, using this algorithm, is not only reliant on a given set of requirements but rather on available data. The program searches for near to full complementarity of conserved 3’UTRs. The alignment across up to 8 vertebrate species is an important consideration in the program to reduce the number of false positives. Once the complementarity and conservation checks are complete, it then uses RNAHybrid to calculate the energy spend of miRNA-3’UTR duplex formation. The program uses energy-cutoffs for different types of binding. Therefore, a seed region with a mismatch may be selected as long as it is within a specified energetic range. Predicted targets are then scored using a Hidden Markov Model (HMM) which is based on a Bayesian classifier model. This statistical model is used to analyse chains or sequences in which the rules governing the production of the chain is not known. By “studying” experimentally validated datasets the model uses a maximum likelihood approach to determine the probability of an observation occurring [2,3].

### Program 3: DIANA-MicroT v3.0 (<http://diana.pcbi.upenn.edu>)

DIANA MicroT v3.0 requires strict seed region base pairing and shows preference for 7mer Watson-Crick paired sites. 6mer sites and seed matches with G:U wobbles are considered if supplementary 3’ binding of miRNA is present, or if binding energy is favourable. Thermodynamic parameters are considered by a 38nt window progressively scanning across a 3’UTR sequence subsequently using a modified dynamic programming approach to calculate free energies of potential binding sites at each step. Conservation is also included in the final scoring, however a non-conserved site may also be considered. Once the target sites are identified they are compared to targets identified from mock sequences and a signal-to-noise ratio and precision score is obtained for each site. Mock sequences are essentially random sequences designed per miRNA that have the same number of seed sites per 3’UTR. These sites are not biologically functional and therefore the ability of the program to recognise the difference allows for the SNR to be determined [4].

## Strategic curation of predicted targets

Relying only on the aforementioned computational programs to identify miRNA targets would be premature. Validation of miRNA targets is a lengthy and relatively expensive procedure and therefore additional analysis and curation of the predicted targets should be completed to filter the potential targets and essentially reduce the number of false positives. In this study, only targets that were predicted across all three programs were considered for the strategic curation developed for this study.

As miR-145, miR-143 and miR-133b seem to commonly be downregulated in CRC, the hypothesis was made that ultimately the combination of the three miRNAs would affect pathways related to the tumourigenesis process and potentially in CRC development. Under this inference the targets identified per miRNA, and that were predicted by all three programs, were loaded into DIANA mirPATH v1, a program that identifies the KEGG pathway enrichment of the combined miRNA predicted targets. KEGG, the **K**yoto **E**ncyclopedia of **G**enomes and **G**enes, is a collection of manually transcribed pathways representing the current knowledge of molecular systems. Once the list of overrepresented pathways were identified, all miRNA targets within these pathways were outlined. Targets linked to more than one CRC identified pathway were weighted more and therefore ranked higher than those only associated with one pathway. Subsequent systemic literature analyses was conducted for all filtered targets to determine a strong reference to CRC, most specifically an increased expression which demonstrates an antagonistic relationship to the downregulated miRNA recognised in CRC.

Supporting Fig 1 illustrates the manual curation methodology used to streamline the selection of potential miRNA targets for further functional analysis.

PicTar

TargetScan 5.1

DIANA-MicroT v 3.0

Hsa-miR-143 (3p and 5p)

Hsa-miR-145 (3p and 5p)

Hsa-miR-133b

DIANA mirPATH

KEGG pathway enrichment

Identify CRC pathways implicated by the predicted targets of the three miRNAs.

List of targets per miRNA within the CRC identified pathways

Through a literature search, identify the targets with the strongest CRCreference

*

Supporting Fig 1: Target prediction methodology and selection of targets for functional analysis. *List of targets within CRC identified pathways were further ranked according to number of associated pathways in addition to the ranking of the pathways by significance.

The mature miRNA sequences for hsa-miR-143-3p, hsa-miR-143-5p, hsa-miR-145-3p, hsa-miR-145p and hsa-miR-133b were submitted into the target prediction algorithms TargetScan 5.1, PicTar and DIANA-Micro T v3.0, as described in section 3.2.1. Overall miR-143 was predicted to have the least number of targets by all three programs, where TargetScan 5.1 predicted 263 conserved targets, PicTar predicted 239 targets and DIANA-Micro T v3.0 predicted 259 target genes. For TargetScan 5.1, there were 272 conserved target sites and 134 poorly conserved target sites within the 263 target genes. Of the 259 genes predicted by DIANA-MicroT v3.0, there were 307 target sites predicted within these genes. MiR-145 yielded large numbers of miRNA targets, most specifically when using TargetScan 5.1 and DIANA-microTv3.0 predicting 528 and 471 target genes. Although PicTar had predicted a lower number of targets for miR-145 compared to the other two computational programs, this number (326) is still higher than the number of target genes predicted for miR-143. TargetScan 5.1 predictions for miR-145 resulted in 585 conserved sites and 174 poorly conserved sites within the 528 target genes. For DIANA-MicroT v3.0 a total of 502 target sites were identified. MiR-133b had also produced large numbers of predicted targets from each program. Some 502 target genes, of which were 530 conserved sites and 80 were poorly conserved sites, were identified from TargetScan 5.1, a total of 471 targets were identified from PicTar and 399 targets (421 target sites) were identified with DIANA-MicroTv3.0.

These results demonstrate that each program yields differential sets of target genes and sites per miRNA. All three programs derive the miRNA mature sequences from mirBase and use RefSeq sequences to map 3’UTRs. The differences in predictions are purely as a result of the different algorithms and weighted scoring and thresholds for the principles of miRNA-target binding considered by each program. The resulting dataset of almost 3500 miRNA targets required further curation and filtering to make the data more meaningful.

The 3458 targets predicted for all miRNAs were then submitted into the DIANA mirPATH v1 algorithm. The resulting KEGG pathways enriched by the combined predicted targets for the three miRNAs were determined. Supporting Fig 2 below represents in histogram format the output of the pathway enrichment. It shows the probabilities of the pathway association when assessing the union of all targets, those targets predicted for each miRNA per target prediction program and also the intersection of the combined targets.


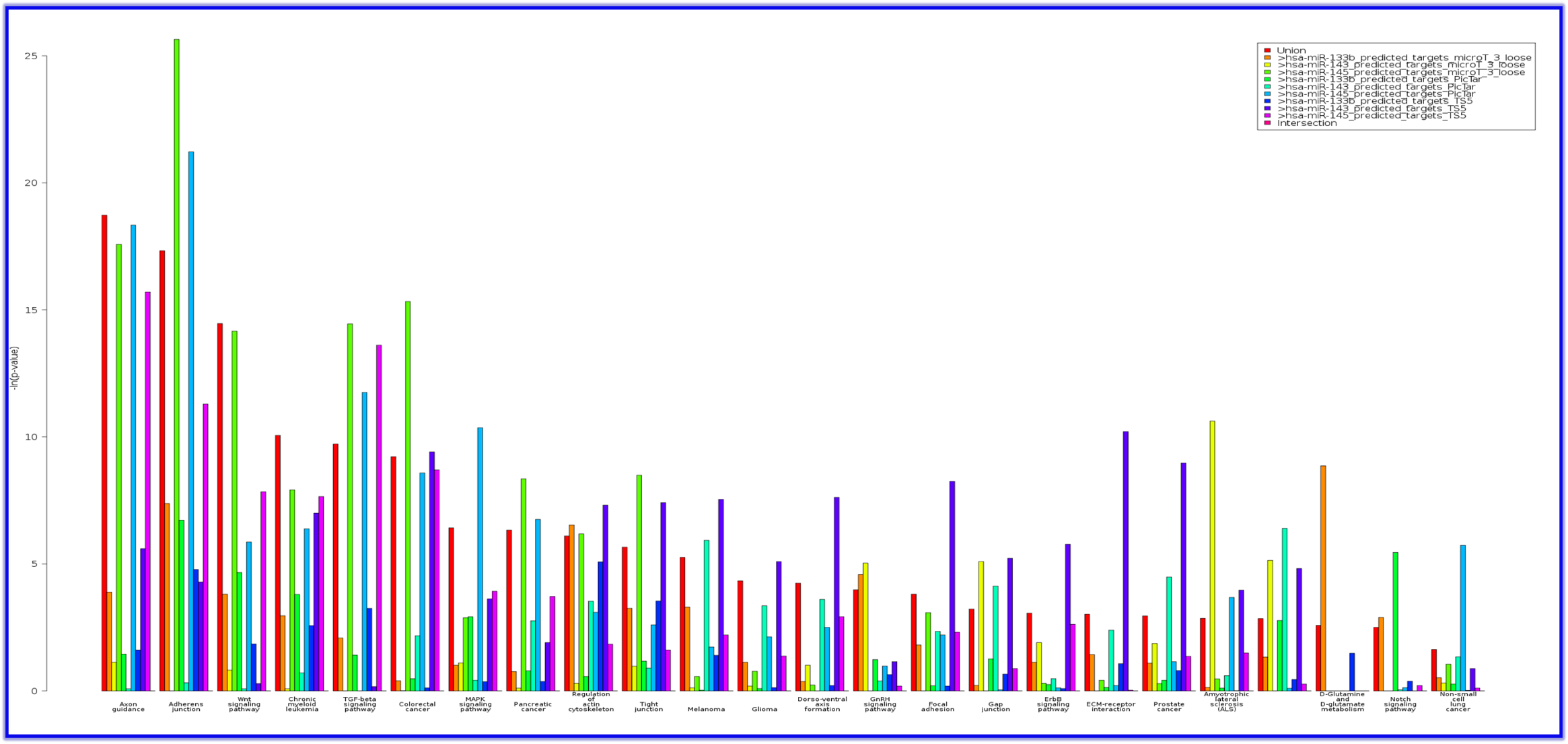


Neurodegenerative disease

Supporting Fig 2: KEGG pathway enrichment of the combined miRNA targets.

miR-143, miR-145 and miR-133b predicted targets from TargetScan, PicTar and DIANA MicroTv3.0 were transferred to DIANA mirPATH to obtain enrichment of KEGG pathways. CRC related pathways were identified to be associated with the combined miRNA targets (circled).

The top 25 significantly enriched pathways from the predicted miRNA targets illustrated in Supporting Fig 2 reveals that pathways potentially influenced by the combined expression of the three miRNAs fall within clearly recognizable categories listed below:

- Cancer (Chronic Myeloid Leukaemia (CML), Colorectal Cancer (CRC), Pancreatic Cancer, Melanoma, Glioma, Prostate Cancer, Non-small cell lung cancer (NSCLC)
- Growth signalling pathways (Wnt signalling pathway, TGF-beta signalling pathway, MAPK signalling pathway and Notch signalling pathway)
- Cell-cell and cell-ECM interactions (Adherens Junctions, Regulation of actin cytoskeleton, Tight junction, Focal adhesion, Gap junction, ECM-receptor interaction)
- Neuron signalling and related disease (Axon guidance, Amyotrophic lateral sclerosis (ALS), Neurodegenerative Disease)

Of particular interest in this study, “Colorectal Cancer” was a KEGG pathway found to be enriched by the combined targets of the three putative tumour suppressor miRNAs. Additionally, several growth signalling pathways previously reported to be deregulated in CRC have also been enriched here with most of them listed within the Top 10 pathways enriched from the combined miRNA predicted targets. This information provides some indication of the involvement of these miRNAs in the development of CRC specifically through these pathways. From these results, one could easily infer that these three miRNAs potentially regulate key CRC-related pathways and could possibly explain the relationship of the miRNAs in the development of CRC. However, as the target prediction programs are essentially just a prediction tool with percentages of false positive rates, these findings require further validation.


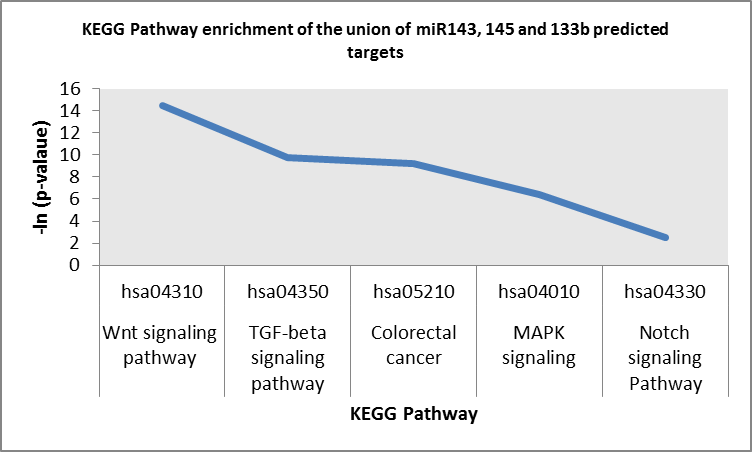


Supporting Fig 3: KEGG Pathway enrichment of CRC-related pathways. Combined targets of miR-143, miR-145 and miR-133b predicted using PicTar, TargetScan and DIANA MicroT. Obtained from DIANA MicroT V1.0.

Based on the DIANA mirPATH v1 output, the CRC-related pathways were ranked for significance according to the probabilities of the pathway being enriched by the miRNA targets (see Supporting Fig 3 above). The Wnt signalling pathway is the most enriched CRC related pathway followed by the TGF-beta signalling pathway, sequentially followed by “Colorectal cancer”, the MAPK signalling pathway and lastly a slight enrichment of the Notch signalling pathway. All predicted targets that fell within these pathways were determined per miRNA and only those targets predicted across all three computational programs were listed for further analysis. Targets falling within more than one CRC-related pathway were ranked higher than those only linked to one; and those linked to a more significantly enriched pathway ranked higher than a less significant pathway. The filtered list of potential miRNA targets are shown in Table 1.

| **Table 1: List of potential targets per miRNA associated with KEGG CRC-related pathways.** | | |
| --- | --- | --- |
| **miRNA** | **Target Gene** | **CRC pathway involvement** |
| miR-143 | **KRAS***  PDGFRA  BCL2*  FGF1  GLI3  CSNK1G3 | **CRC, MAPK**  CRC, MAPK  CRC  MAPK  NOTCH  NOTCH |
| miR-145 | SMAD3*  **FZD7**  PPP3CA*  CTNNBIP  CCND2  ZFYVE9  INHBB  FLNB  RASA2  DUSP6  RASA1 | TGF-beta, Wnt, CRC  **Wnt, CRC**  Wnt, MAPK  Wnt  Wnt  TGF-beta  TGF-beta  MAPK  MAPK  MAPK  MAPK |
| miR-133b | *PPP2CB  *PPP2CA  **FBXW11**  NFAT5  SP1*  EVI1  FGFR1*  MAP3K3  CRK  CSNK1G3 | TGF-beta, Wnt  TGF-beta, Wnt  **Wnt, NOTCH**  Wnt  TGF-beta  MAPK  MAPK  MAPK  MAPK  NOTCH |

*Experimentally validated targets

A systemic literature search was conducted on each predicted target represented in Table 1. The search involved assessing gene or protein expression in CRC and their potential roles in CRC development. Most importantly however, evidence of oncogenic potential of the target was assessed. If the predicted gene is a true target of the miRNAs in question, then upon downregulation of the specific miRNAs, one could expect to see a resultant increased expression of the target in CRC. After the literature search was completed, the targets boxed in red in Table 3.2 were chosen for functional analysis due to their strong inference of an increased expression in CRC. A detailed view of the putative miRNA-target binding sites is described further.

Three sites in the KRAS gene (ENSG00000133703) were predicted by all three programs as miR-143 targets. Two 8mer sites were conserved across several vertebrate species. A 7mer-1A site demonstrated poor conservation amongst aligned vertebrate orthologues. MiR-143-KRAS binding sites are depicted in Supporting Fig 4:


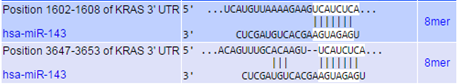
Conserved sites:


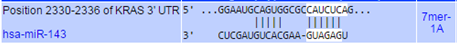
Poorly conserved site:

Supporting Fig 4: miR-143 binding sites in the KRAS gene. Two 8-mer sites were predicted with the seed regions positioned at region 1602-1608 and 3647-3653 of the KRAS 3’UTR.

Within the FZD7 (ENSG00000155760) gene chosen as a potential miR-145 target for further functional analysis, a single 7mer-m8 site was predicted across all three computational programs. The seed region of the site is located at position 518-524 of the FZD7 3’UTR. MiR-145-FZD7 putative binding is depicted in Supporting Fig 5 below.


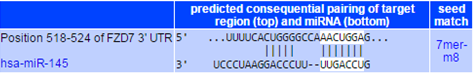


Supporting Fig 5: miR-145 binding site in the FZD7 gene. A single 7mer-m8 site was predicted with the seed region positioned at region 518-524 in the FZD7 3’UTR.

For miR-133b, the FBXW11 (ENSG00000072803) gene was selected as a putative target for further functional analysis. A single 7mer-m8 site wherein the seed region is located at position 1622-1628 of the FBXW11 3’UTR was predicted across all three computational target prediction programs used in this study. The miR-133b-FBXW11 binding site is depicted below in Supporting Fig 6.


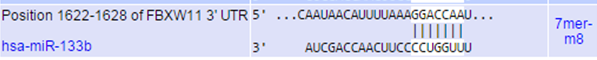


Supporting Fig 6: miR-133b binding site in the FBXW11 gene. A single 7mer-m8 site was predicted with the seed region positioned at 1622-1628 in the FBXW11 3’UTR.

*In silico* miRNA target prediction provided a platform to identify potential targets for each miRNA and the chosen targets were supported by scientific literature in relation to CRC development. In order to test the functionality of these potential targets, the protein levels were measured by immunofluorescence, before and after HET1a cells were transfected with Anti-miRs.

# RNA extraction

## 2.1 Total RNA Isolation

RNA extractions were performed using the RNeasy Plus Mini kit (Qiagen) for cDNA synthesis and primer optimisation, as well as the Nucleospin® RNA II kit (Separations - Macherey Nagel) for final experiments.

## 2.2 RNeasy plus mini kit

This kit is designed to purify RNA and has an additional step which eliminates whole genomic DNA contamination, which is essential for relative quantification of gene expression.

RNA was isolated following the prescribed protocol from Qiagen. A 5mL solution of RLT buffer was prepared and supplemented with reducing agent β-mercaptoethanol (β-ME) (Sigma-Aldrich) at 10μL/mL. RLT buffer allows for the disruption of plasma membranes of cells and organelles to release all RNA contained in the sample. Incomplete disruption may result in inefficient lysis and reduced RNA yields. 600μL of β-ME/RLT buffer was added to the pellet and this was resuspended under a fume hood. The lysed cells were then frozen at -70°C. RNA is stable for 7 months after the addition of β-ME/RLT buffer. When ready to proceed these lysates were thawed out at room temperature (25°C) before the next step.

A 600μL volume (maximum volume 700μL) of cell lysate was pipetted directly into a QIAshredder spin column placed in a 2mL collection tube and centrifuged at 14 000rpm for 2 minutes. The homogenised lysate was then transferred to a gDNA Eliminator spin column placed in a 2mL collection tube and subsequently centrifuged at 10 900rpm for 30 seconds. The column was discarded and the flow-through was collected. One volume of refrigerated 70%v/v ethanol (600μL) was added to the flow-through and mixed well by pipetting.

A maximum of 700μL of the sample was transferred each time to an RNeasy spin column placed in a 2mL collection tube until the entire 1200μL volume was transferred (to maximise RNA yields). Samples were centrifuged at 10 900rpm for 15 seconds after which the flow-through was discarded and the column was retained.

700μL of buffer RW1 was added to the RNeasy column and samples were centrifuged at 10 900rpm for 15 seconds to wash the spin column membrane. The flow-through was discarded and the column was retained.

500μL of buffer RPE was added to the RNeasy column and samples were centrifuged at 10 900rpm for 15 seconds to wash the spin column membrane. The flow-through was discarded and the column was retained.

500μL of buffer RPE was added to the RNeasy column and samples were centrifuged at 10 900rpm for 2 minutes to wash the spin column membrane. The flow-through was discarded and the column was retained. The longer centrifugation step allows the spin column membrane to dry ensuring that no ethanol is carried over during RNA elution as downstream reactions may be hindered by the presence of residual ethanol.

The RNeasy spin column was then carefully removed from the collection tube to prevent contact with the flow-through, thus preventing the carry-over of ethanol. The RNeasy spin column was placed into a new 2mL collection tube and centrifuged at 14 000rpm for 1 minute. The RNeasy spin column was subsequently placed into a new 1.5mL collection tube, and 30μL of RNase-free water was added directly to the spin column membrane. The sample was incubated at room temperature (25°C) for 5 minutes and was then centrifuged thereafter at 10900rpm for 1 minute to elute the RNA. The RNA was subsequently quantified.

## 2.3 Nucleospin® RNA II kit

The Nucleospin® RNA II kit allows for the purification of RNA and isolates RNA of high integrity, reducing the risk of degradation and DNA contamination as a result of the on-column digestion with rDNase.

RNA was isolated following the protocol provided by Macherey-Nagel. Up to 5 x 106 cells could be pelleted and lysed by 350μL of buffer RA1 supplemented with 3.5μL of reducing agent β-ME. A total volume of 353.5μL of β-ME/RA1 was added to the cell pellet and vortexed vigorously. Cells were frozen at -70°C as RNA is stable for 7 months after the addition of β-ME/RA1 buffer. When ready to proceed, these lysates were thawed out at room temperature (25°C) before the next step.

The entire quantity of lysate was filtered through a Nucleospin® filter placed in a collection tube to reduce viscosity, and the mixture was centrifuged at 11000rpm for 1 minute. The Nucleospin® filter was discarded and 350μL ethanol (70%v/v) was added to the homogenised lysate to adjust RNA binding conditions. This was mixed by pipetting up and down about five times or by vortexing for about 5-10 seconds. The addition of ethanol leads to a stringy appearance of the solution, which needs to be disaggregated by mixing.

The total volume of ethanol was passed through a Nucleospin® RNA II column placed in a collection tube. Once the lysate was thoroughly mixed, it was loaded to the column and centrifuged for 30 seconds at 11000rpm to allow the binding of RNA to the column.

The column was placed in a new collection tube to which 350μL of membrane desalting buffer (MDB) was added and this was centrifuged at 11000rpm for 1 minute to allow the membrane to dry. Salt removal allows for more efficient DNA digestion.

The rDNase provided in a vial was reconstituted with 540μL RNase-free water. A DNase reaction mixture was prepared in a sterile 1.5mL tube where 10μL of reconstituted rDNase was added to 90μL of reaction buffer for rDNase and was mixed by flicking the tube. Subsequently, 95μL of this DNase reaction mixture was added directly to the centre of the silica membrane of the column. This was incubated for 15 minutes at room temperature (25°C).

The silica membrane was subsequently washed and dried through three washes. The initial step involved the addition of 200μL buffer RA2 to the Nucleospin® RNA II column in order to inactivate the rDNase. This was centrifuged at 11000rpm for 30 seconds. The column was then placed in a new collection tube, to which 600μL of buffer RA3 was added to the column to wash away any residual buffer added to the column in the previous wash, and this was centrifuged at 11000rpm for 30 seconds. The column was once again placed in a new collection tube to which 250μL of buffer RA3 was added to wash away all residual buffer that may still be present on the column; and to dry the membrane completely, this was centrifuged at 11000rpm for 2 minutes. The Nucleospin® RNA II column was then placed in a nuclease-free collection tube.

The last step involved the addition of 40μL RNase-free water to the column and this was incubated at room temperature (25°C) for 5 minutes to allow absorption into the membrane. The sample was then centrifuged at 11000rpm for 1 minute. Elution in a smaller volume of water allows a higher yield of RNA, thus 40μL was used instead of the recommended 60μL. The RNA was subsequently quantified.

## 2.4 RNA quantification

The quality of RNA was then assessed using a Nanodrop ND-1000 spectrophotometer which measures the optical density of the sample RNA. RNA of good quality displays an OD 260/280 ratio of 1.8 to 2.1 (a ratio below this indicates protein contamination and a ratio above this suggests the RNA has degraded) and an OD 260/230 ratio of 1.8 or greater (a ratio below this signifies salt contamination). The ND-1000 also assesses the concentration of RNA within a sample where an optimal concentration of 1000ng/μL or greater is generally used for relative quantification.

## 2.5 miRNA PCR amplification

Real time PCR proceeded using a TaqMan® Universal PCR Master Mix and TaqMan® MicroRNA Assays (Applied Biosystems). TaqMan® technology utilises the basic concepts of polymerase chain reaction (PCR) and fluorescence resonance electron transfer (FRET) in combination to yield a superior version of the conventional PCR, more accurately amplifying the target being investigated. MiRNA expression was normalised to the housekeeping non-coding RNA 18s rRNA and detected relative to no treatment controls in a 7500 Real Time PCR Machine (Applied Biosystems). Input cDNA concentrations were standardized prior to experimental runs on each cell line. MiRNA-specific TaqMan® MGB probes and primers were used to detect expression of the respective miRNAs. Samples were run in triplicate to establish a mean Ct value for the amplification of each sample. The parameters programmed into the 7500 Real Time PCR machine for each run is shown in Supporting Fig 7 below.

| **Step** | **AmpliTaq Gold Enzyme Activation** | **Polymerase Chain Reaction** |
| --- | --- | --- |
|  | HOLD | CYCLE (40 cycles) |
|  |  | Denature Anneal/ Extend |
| **Time** | 10 min | 15 sec 60 sec |
| **Temperature (°C)** | 95 | 95 60 |

Supporting Fig 7: Parameters programmed in the Applied Biosystems 7500 Real Time PCR Machine for each run.

The housekeeping non-coding RNA 18SrRNA was amplified using an assay containing the specific primers and probes targeting this gene. This assay was commercially available through Applied Biosystems.

## 2.6 Data analysis

Sample reactions were run in triplicate. The mean Ct values were determined during the experimental runs and subsequently the miRNA expression levels from the treated samples were first normalised to 18SrRNA and then calculated relative to no treatment controls, according to the 2^-ΔΔCt^ method described by [5] (see 2.6.1).

To compare the relationship between the sample means before and after treatment with the epigenetic treatments, a paired two-tailed Student’s t-test was performed with the confidence interval set at 95%.

### 2.6.1 The 2^-ΔΔCt^ method

The 2**^-ΔΔCt^** method was used to assess relative gene expression in this study [5]. The Ct of the target gene was first normalised to that of the reference gene (in this case 18S rRNA) for both test and control samples following equations:

ΔCt(miR) = Ct (miR - treated) − C(18s rRNA - treated)

ΔCt(control) =Ct (miR - control) − Ct (18s rRNA - control)

The CT of the miR sample was then normalised to the CT of the control as seen below:

ΔΔCt = ΔCt(miR) −ΔC(control)

As a result, the ratio of the target gene in the test sample to the calibrator sample is found, which is normalised to the reference gene. All treated sample results are relative to the untreated samples. The expression ratio can be calculated as below:

Normalised target gene expression = 2**^(-ΔΔCt)^**

References:

1. Lewis, B.P., Burge, C.B., Bartel, D.P. (2005) Conserved Seed Pairing, Often Flanked by Adenosines, Indicates that Thousands of Human Genes are MicroRNA Targets. *Cell*. **120(1)**: 15-20
2. Krek, A., Grün, D., Poy, M.N., et al. (2005) Combinatorial microRNA target predictions. *Nat Genet*. 3**7(5)**:495-500
3. Lall, S., Grün, D., Krek, A., et al. (2006) A genome-wide map of conserved microRNA targets in C. elegans. *Curr Biol*. **16(5)**:460-471
4. Kiriakidou, M., Nelson, P.T., Kouranov, A., et al. (2004) A combined computational-experimental approach predicts human microRNA targets. *Genes Dev*. **18**:1165–1178
5. Livak, K.J., Schmittgen, T.D. (2001) Analysis of Relative Gene Expression Data Using Real-Time Quantitative PCR and the 22DDCT Method. *Methods*. **25**, 402–408
